# Factors impacting on the decision of graduate entry medical school students to pursue a career in obstetrics and gynecology in Ireland

**DOI:** 10.1186/s12909-023-04425-8

**Published:** 2023-06-19

**Authors:** Eimear Spain, Mary-Elizabeth Tumelty, Ailish Hannigan, Kaitlyn Cinnamond, Ayesha Cheema, Amanda Cotter

**Affiliations:** 1 School of Medicine, University of Limerick, Limerick, Ireland; 2 School of Law, University College Cork, Cork, Ireland; 3 University Hospital Limerick, Limerick, Ireland; 4 Nottingham University Hospitals NHS Trust, Nottingham, UK; 5 University Maternity Hospital Limerick, Limerick, Ireland

**Keywords:** Career, Obstetrics, Gynaecology, Rotation, Recruitment, High-risk, Work-life balance

## Abstract

**Background:**

Challenges in recruiting appropriately trained obstetricians and gynaecologists have been identified across the world. Given well documented staff shortages within obstetrics and gynaecology in Ireland, it is increasingly important to understand the factors which influence medical students to choose or reject a career in the speciality. The aim of this study was to ascertain the perceptions of final year graduate entry medical students of obstetrics and gynaecology, including the factors which may influence a student’s decision to pursue in a career in the speciality.

**Methods:**

Paper-based surveys of graduate entry medical students (n = 146) were conducted at the beginning and end of a six week rotation in obstetrics and gynaecology in Ireland. Responses to the surveys pre- and post-rotation were matched and changes in career choices, merits and demerits over time were analysed. All analysis was conducted using SPSS for Windows version 25.

**Results:**

The responses of 72 students to both questionnaires could be matched (response rate of 49.3%). No male students expressed an interest in obstetrics, gynaecology or both as a first choice of career in the pre rotation survey. Obstetrics as a first choice of career increased from 6.9% pre rotation to 19.4% post rotation (p = 0.04) and this increase was seen in male and female students. Gynaecology as a first choice increased slightly from 1.4 to 4.2% (p = 0.50) and the dual speciality increased from 6.9 to 13.9% (p = 0.23). Students identified many merits of obstetrics pre-rotation with more than 60% identifying it as exciting, interesting fulfilling and challenging. However, incompatibility with family life was cited as a demerit by 72% of respondents and 68.1% identified fear of litigation as a demerit. Participants were less positive overall about the merits of a career in gynaecology with less than 40% viewing it as exciting, fulfilling, and varied.

**Conclusions:**

While respondents were positive about the merits of a career in obstetrics and gynecology, concerns remain about work-life balance, career opportunities, and the high-risk nature of the specialty. These concerns should be addressed by the profession and policy makers if they wish to attract sufficient numbers to address anticipated need in the coming years. Gender differences in speciality choice were also evident. If males are to be recruited into obstetrics and gynaecology, consideration should be given to the positive impact of internship.

**Supplementary Information:**

The online version contains supplementary material available at 10.1186/s12909-023-04425-8.

## Background

Difficulties in identifying appropriately trained obstetricians and gynecologists have been documented around the world, in countries including the US, Oman, and Saudi Arabia [1–4]. Staff shortages can cause existing staff to be overworked and put women and their babies at risk. Mahha et al. have recognised this as being a contributory factor in poorer patient outcomes, including the loss of pregnant women and their babies [4–6].

In Ireland, the shortage of appropriately trained consultant obstetricians and gynaecologists has been acknowledged for many years [7–9]. Ireland has the lowest number of consultant obstetricians and gynaecologists per 100,000 females amongst wealthy OECD countries [7, 8] with 3.6 per 100,000 in Ireland compared to 4.6 in England, 4.7 in Scotland, 6.6 in Australia and 6.2 in New Zealand [7]. This is compounded by the challenges faced due to retirement in the short to medium term with 40% of consultants in the speciality in Ireland aged 55 years or over [9]. In a review of consultant needs to 2028 and the training pipeline needed to meet this demand, it was estimated that an increase of 50% in the number of current obstetricians and gynaecologists is required to meet anticipated demand in Ireland [9]. Retention may also prove to be an issue with a survey of doctors in training working in obstetrics and gynaecology reporting that 71.5% indicated that they had considered leaving the speciality [10].

In the UK, the Centre for Workforce Intelligence reported a 53% increase in the consultant workforce between 2003–2013 [11]. Despite this, it was reported in 2017 that “88% of obstetric units reported difficulties in filling middle-grade rotas” [12, 13]. More recently, Ismail and Kevelighan described the speciality as facing a “recruitment crisis with < 1 in 30 of medical school graduates seeking the speciality as their first career choice” [14] .Recruitment is therefore an issue worthy of consideration by the profession in many jurisdictions, not least because of patient safety concerns.

In this context, the attractiveness of the speciality to graduates and the availability of training becomes very significant. Research indicates that the numbers who seek to pursue obstetrics and gynecology as a career are low nationally and internationally [4, 12, 15–17]. However, difficulties extend beyond recruiting doctors to the speciality, with a recent study by the Royal College of Obstetricians and Gynaecologists raising concerns about high attrition rates from training programmes, specialists retiring early or leaving practice [18].

Against this backdrop, the objective of this study was to explore for the first time the views of graduate entry medical students in Ireland on obstetrics and gynaecology as a career choice, and the factors which influence their decision on whether to pursue a career in the speciality.

## Methods

University of Limerick Graduate Level Entry Medical School is unique in Ireland as it is the only one of Ireland’s six medical schools to accept only graduate entry students into its four year programme. Three other institutions also offer graduate entry programmes, in addition to their undergraduate degrees. Graduate entry students have different levels of maturity, learning style and career plans [19–21] and this research sought to ascertain the perception of obstetrics and gynaecology amongst this growing cohort of medical students. Medical students are required to complete a six week intensive teaching rotation in obstetrics and gynaecology in their final year in University Maternity Hospital Limerick. University Maternity Hospital Limerick is the sole provider of obstetric, midwifery and neonatology services in the Midwest region. In 2020, there were 4,149 live births. To specialise in obstetrics and/or gynaecology in Ireland one must complete training in both obstetrics and gynaecology.

### Participants

All fourth year graduate entry medical students in the University of Limerick (n = 146, 61% female) were invited to participate at two time points; day one of their rotation and six weeks later, upon completion of their rotation in obstetrics and gynecology in the University Maternity Hospital, Limerick.

### Measures

Two paper-based surveys (a 15 item pre- and 19 item post- rotation questionnaire) were developed by researchers based in the University of Limerick, Ireland. No existing surveys addressed our domains of interest at the time of this study. Design of the survey questionnaire was therefore informed by the literature and some previously validated questions [20, 21]. The survey included questions on demographics, views on a career in obstetrics and/or gynaecology, perceived merits and demerits of the specialties, and the impact of the regulatory and medico-legal culture on career choices. Both surveys were broadly similar to allow an exploration of the impact of the rotation on participants. Four additional questions were added to the questionnaire administered after the rotation to capture the full experience on placement.

#### Ethical approval

was obtained from the Faculty of Education and Health Sciences Research Ethics Committee at the University of Limerick. A gatekeeper was used to distribute, collect and remove any identifying data before the researchers involved in supervision and grading gained access to it.

### Statistical analysis

Categorical variables are summarised using counts and percentages. Numeric data was tested for normality and median (range) used for non-normal distributions. McNemar’s test was used to test for differences in paired nominal data pre and post rotation. A 5% level of significance was employed for all statistical tests with no adjustment for multiple testing. All analysis was undertaken using SPSS for Windows Version 25.

## Results

Responses from 72 students (response rate 49%) could be matched pre and post rotation and therefore, only the responses of 72 students were included in the data analysis. The characteristics of these participants are summarised in Table 1.

**Table 1:** Characteristics of participants (n = 72)^1^

Characteristic	Frequency (%)
Gender	
Male	24 (33.3%)
Female	47 (65.3%)
Prefer not to say	1 (1.4%)
**Age**	
Median (min, max)	26 (24–34)
**Type of Primary Degree**	
Health Related	51 (71.8%)
Non-Health Related	20 (28.2%)
**Time since obtaining primary degree** ^1^	
≤ 3 years	15 (21.1%)
4–5 years	40 (56.3%)
> 5 years	16 (22.5%)

^1^Missing data for up to 3 participants for some variables; percentages of valid responses given

The majority of participants (65%) identified as female, which is similar to the full cohort (61%). The median age was 26 years (range 24–34 years) and the majority (71.8%) had a primary degree in a health-related discipline.

### Interest in obstetrics and/or gynaecology as a career generally

When asked if they were interested in a career in obstetrics, gynaecology or both, five (6.9%) participants identified obstetrics as their first choice pre rotation, with 12 (16.7%) identifying it as a second choice. Gynaecology was less attractive with just one (1.4%) and six (8.3%) participants interested in it as a first or second choice respectively. Five (6.9%) were interested in the dual specialism as a first choice, and eight (11.1%) were interested in it as a second choice. No male students expressed an interest in obstetrics, gynaecology or both as a first choice of career in the pre rotation survey.

Interest amongst this cohort in a career in obstetrics, gynaecology or both as a first choice increased following the rotation. The biggest increase was for obstetrics as a first choice (6.9–19.4%, p = 0.04) and this increase was seen in male and female students (Table 2). Gynaecology as a first choice increased slightly from 1.4 to 4.2% (p = 0.50) and the dual speciality increased from 6.9 to 13.9% (p = 0.23).

**Table 2:** Interest in obstetrics, gynaecology or both as a first choice of career by gender and pre and post rotation

	Obstetrics		Gynaecology		Both	
	Pre	Post	Pre	Post	Pre	Post
1st choice (% Total)	5 (6.9%)	14 (19.4%)	1 (1.4%)	3 (4.2%)	5 (6.9%)	10 (13.9%)
Female (% Female)	5 (10.6%)	11 (23.4%)	1 (2.1%)	3 (6.4%)	5 (10.6%)	9 (19.1%)
Male (% Male)	0 (0%)	3 (12.5%)	0 (0%)	0 (0%)	0 (0%)	1 (4.2%)

### Merits of a career in obstetrics

Participants identified many merits of a career in obstetrics pre rotation, with the majority viewing it as exciting (65.3%), interesting (77.8%), fulfilling (77.8%), and challenging (66.7%). Additionally, a minority viewed obstetrics as varied (41.7%) and/or having good training and career opportunities (25%).

**Fig. 1:**
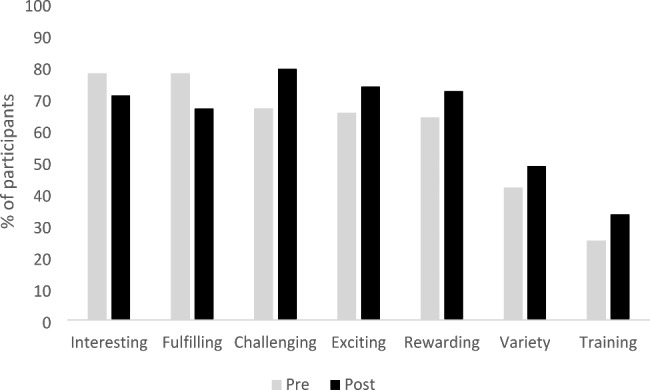
Perceptions of merits of a career in obstetrics pre and post rotation (n = 72)

Figure 1 shows the perception of each merit pre and post rotation. The percentage who perceived the career as exciting, challenging, rewarding, varied and having good training and career opportunities increased post rotation but there were decreases in the percentage who perceived it as interesting or fulfilling. The biggest change was in the percentage of participants who viewed it as a challenging career, increasing from 66.7% pre rotation to 79.2% post rotation.

### Merits of a career in gynaecology

**Fig. 2:**
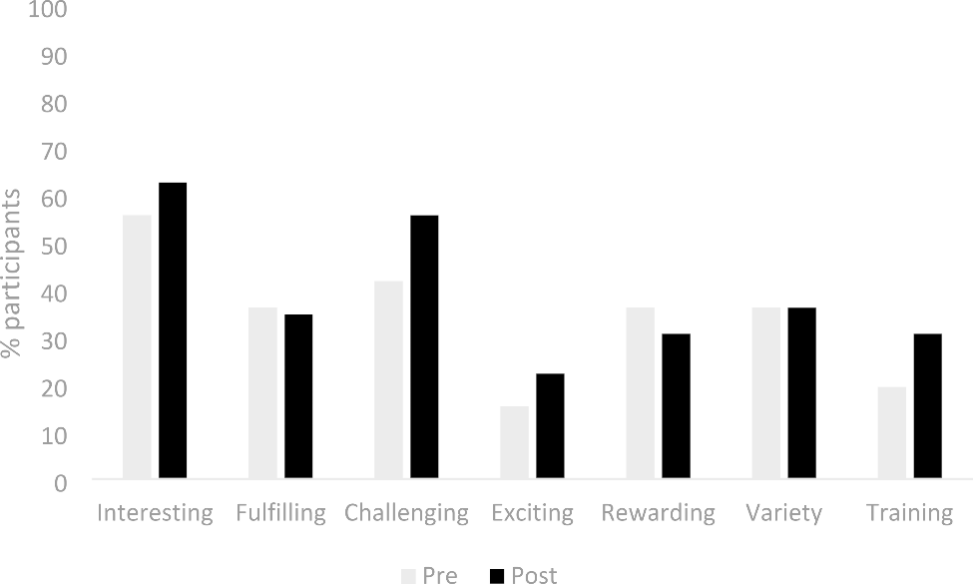
Perceptions of merits of a career in gynaecology pre and post rotation (n = 72)

Participants were less positive overall about the merits of a career in gynaecology pre rotation with just 15.3% viewing it as exciting, 36.1% as fulfilling, 36.1% as varied, 41.7% viewed it as challenging and 19.4% identifying training/career as a merit of the specialty before their rotation. Figure 2 shows the perception of each merit pre and post rotation. The percentage who perceived the career as exciting, challenging, interesting and having good training and career opportunities increased post rotation but there were decreases in the percentage who perceived it as rewarding or fulfilling. The biggest change was in the percentage of participants who viewed it as a challenging career, increasing from 41.7% pre rotation to 55.6% post rotation.

### Demerits of a career in obstetrics

**Fig. 3:**
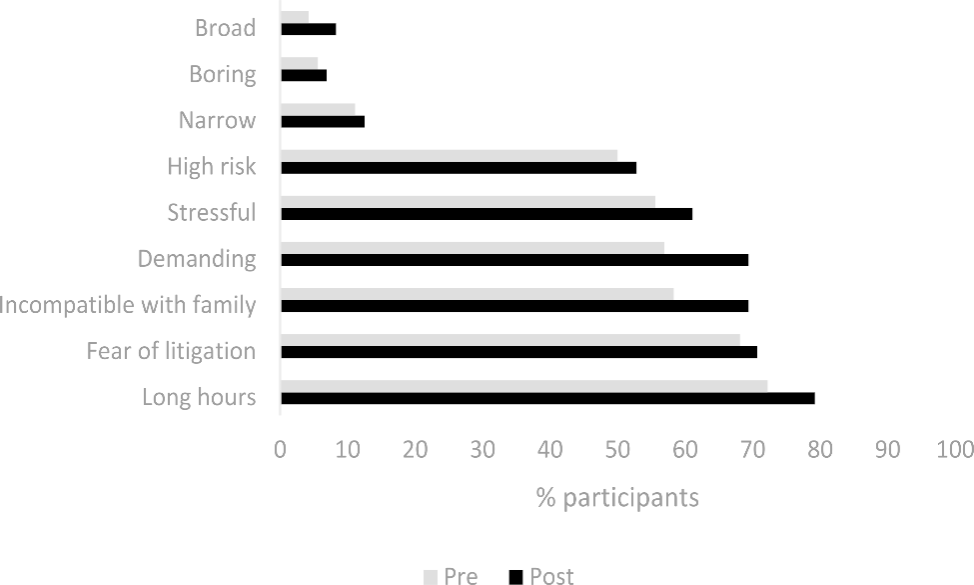
Perceptions of demerits of a career in obstetrics pre and post rotation (n = 72)

Data was also collected on the perception of participants of the demerits of a career in obstetrics or gynaecology. Pre rotation participants viewed obstetrics as demanding (56.9%), stressful (55.6%), having long hours/being incompatible with family life (72.2%) and high risk (50%) while 68.1% identified a fear of litigation as a demerit. Interestingly, only 5.6% viewed the speciality as boring, 11.1% as narrow and 4.2% as broad. Figure 3 shows the perception of each demerit pre and post rotation. Percentages for all demerits increased post rotation with the biggest increase for the perception of a demanding career, increasing from 56.9% pre rotation to 69.4% post rotation.

### Demerits of a career in gynaecology

**Fig. 4:**
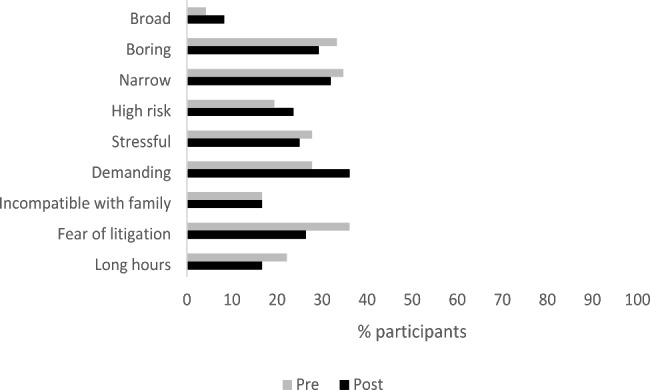
Perceptions of demerits of a career in obstetrics pre and post rotation (n = 72)

The demerits of gynaecology were less obvious with a minority viewing it as demanding (27.8%), boring (33.3%), stressful (27.8%), having long hours/being incompatible with family life (22.2%), high risk (19.4%), narrow (34.7%), broad (4.2%) while 36.1% identified a fear of litigation as a demerit. Figure 4 shows the perception of each demerit pre and post rotation. Changes pre and post were small (< 10%) with the biggest change for fear of litigation decreasing from 36.1% pre rotation to 26.4% post rotation.

### Factors impacting on the decision to pursue a career in OBGYN

The factors that may impact a participant’s decision to pursue a career in obstetrics and/or gynaecology more specifically were also explored. It is clear that the intensity of the workload is a factor in the decision to pursue a career in obstetrics with 64.3% of participants indicating that they were somewhat or to a great extent influenced by this factor compared to 46.4% for gynaecology. A minority of participants (45%) indicated that having to pursue both obstetrics and gynaecology impacted on their decision to pursue obstetrics somewhat or to a great extent, compared with 40% in relation to gynaecology. The results show similar patterns pre and post rotation.

## Discussion

Despite very positive perceptions about the merits of a career in obstetrics, just five (6.9%) participants identified obstetrics as their first choice pre rotation. Gynaecology was less attractive with just one (1.4%) interested in it as a first choice. However, both obstetrics and/or gynaecology became more attractive as a first choice post rotation, with obstetrics more than doubling in popularity to 19.4% and 13.9% interested in the dual specialism of obstetrics and gynaecology as a first choice. Interest in gynaecology only as a first choice, while increasing from a very low base, remained low at 4.2%, demonstrating a clear preference amongst students for obstetrics [14].

These figures are significantly higher than international comparators. A survey conducted in Switzerland showed that 5.9% of fourth year residents were interested in a career in Obstetrics and Gynaecology [22]. Large scale studies conducted in the UK from 1975 to 2005 demonstrated a decline in obstetrics and gynaecology as a first choice in the 1990s “from 4.2% of 1996 qualifiers to 2.2% of 1999 qualifiers” [17]. A 2012 UK study identified 3.8% of respondents having chosen obstetrics and gynaecology as a career, with 8.7% found to have seriously considered but subsequently rejected the speciality [16]. Subsequent studies identified the speciality as “the first choice of career for 5.7% of post-2002 graduates in year 1, 4.3% in year 3 and 3.8% in year 5.” [12]. In two studies of graduate entry medical students in Wales published in 2014 and 2020, Ismail and Kevelighan found that 4% and 3.9% would consider the speciality as first career choice respectively [14, 21]. Interestingly, in the latter study 61.3% would consider it as a second option, demonstrating the importance of addressing student concerns about the speciality and harnessing their enthusiasm as they complete medical school [12, 14]. The low interest levels in obstetrics and gynaecology internationally are a concern given the need to increase recruitment into the specialism(s) and leads to considerations of why the speciality is not more attractive. In an Irish context, the increased interest post-rotation is encouraging, placing us in a better position than colleagues internationally to meet future demand and leading to considerations of the availability of training opportunities to meet demand amongst this cohort.

The results of this research echo previous national and international findings which indicate that individuals identifying as female are more likely to be interested in the speciality [4]. Interestingly, individuals identifying as male were more likely to pursue this specialism post-rotation. This is reflective of the positive impact of the rotation on perceptions of obstetrics and gynaecology more generally, highlighting that students “can be persuaded to reconsider obstetrics and gynecology as a future career” [4, 21] .Continued exposure to the specialism will therefore be vital to recruitment efforts in the future [21].

The factors impacting upon the popularity of obstetrics are varied, with work life balance an oft-cited concern, including almost 80% of the respondents to this survey who viewed obstetrics as having long hours and/or being incompatible with family life post rotation. 62.5% of respondents to our survey indicated that the intensity of the workload is a factor in the decision of whether to pursue a career in obstetrics and 44.4% when contemplating gynaecology. O’Sullivan notes that, the speciality is “traditionally perceived as a “lifestyle unfriendly” [23]. Stokes et al. suggest that it is highly likely that “controllable lifestyle is at the forefront for many trainees” in obstetrics and gynaecology [24]. A 2010 study of over 600 medical students in Australia found obstetrics and gynaecology ranked second from the bottom of 19 specialities in terms of lifestyle friendliness [25]. In a study of specialities which were seriously considered by doctors but subsequently rejected published in 2012, work life balance was the most oft cited reason for rejecting obstetrics and gynaecology [16]. So while the merits of obstetrics and gynaecology are appreciated by medical students, concerns around work life balance must be addressed if the speciality is to meet future staffing demands. Research has found that one of the few differences between graduate and direct entry medical students is in relation to the importance placed on ‘quality of life’, with family time viewed as more important by graduate entrants [26]. This may also explain why graduate entrants have been found to prefer general practice to hospital specialities [27, 28]. Despite the fact that the differences identified in both studies were small in scale, it is something to be considered in the recruitment of graduate students.

Fear of litigation was identified as a demerit of both obstetrics and gynaecology, reflective of the growth in medical negligence litigation in Ireland, with more than 1,000 medical negligence actions commenced annually in the Irish High Court [29]. Just one third of respondents considered career opportunities and training to be a merit of obstetrics or gynaecology post rotation. This is an issue which should be addressed given previous research which indicated that poor career prospects was a significant factor in the rejection of obstetrics and gynaecology as a career [16, 30]. As Turner et al. note, “[t]he unwillingness of young doctors to enter obstetrics and gynaecology may be attributable to concerns about workforce planning and career progression problems, rather than any lack of enthusiasm for the specialty.” [17] This is something which should be considered, including why men felt less positive about career prospects and training opportunities and what steps can and should be taken to correct this.

International studies have focused on perceptions in obstetrics and gynaecology at different points in time including during placements [21], at the end of placements [31] and after graduation [16, 17, 22]. The ratings in this study were obtained in the immediate aftermath of a placement in obstetrics and gynaecology and the views and priorities of participants may change following exposure to other specialties either during their undergraduate studies, intern year or beyond. Follow up studies following graduation may illuminate if the views expressed are settled or open to change over time.

## Conclusion

With an aging workforce and already understaffed service, Ireland faces serious challenges to recruit and retain young doctors in obstetrics and gynaecology. As has been previously discussed, this challenge is not unique to Ireland. It is therefore critical to understand how the speciality is viewed amongst medical students and the challenges it faces in recruiting the next generation of obstetricians and gynaecologists. The research has highlighted the very positive role played by the placement in student perceptions of, and interest in, a career in obstetrics and gynaecology. Increasing exposure to the specialty in the medical curriculum could prove to be a key mechanism to harness student interest and increase recruitment to obstetrics and gynaecology. This research provides new insights into the factors which influence the decision of graduate entry medical students to pursue or reject a career in the speciality. In doing so, it provides insights into the decision-making process of graduate doctors for the first time in an Irish context, whilst contributing to the international debates on this topic. Information of this nature will help encourage an understanding of barriers to increased recruitment and retention in obstetrics and gynaecology.

## Electronic supplementary material

Below is the link to the electronic supplementary material.

Supplementary Material 1

Supplementary Material 2

## Data Availability

The datasets used and/or analysed during the current study available from the corresponding author on reasonable request.
