# Supplementary material for: Factors impacting on the decision of graduate entry medical school students to pursue a career in obstetrics and gynecology in Ireland
Source: BMC Med Educ. 2023 Jun 19;23:449. doi: 10.1186/s12909-023-04425-8 (PMC10278328; doi:10.1186/s12909-023-04425-8)
Supplement: Supplementary file 2 — Supplementary Material 2 [file 12909_2023_4425_MOESM2_ESM.pdf]

UNIVERSITY of LIMERICK
OLLSCOIL LUIMNIGH

Questionnaire post-rotation

Please provide the last four digits of your UL ID number to allow us to match your responses pre and post completion of your rotation in obstetrics and gynecology

____

1. Age (years) ____________________

2. Time since obtaining primary degree (years)

1 year	
2 years	
3 years	
4 years	
5 years	
6 years	
7 years	
8 years	
9 years	
10 or more years	

3. Type of primary degree

Health related	
Non health related	

4. Gender

Male	
Female	
Prefer not to answer	

5. Following your rotation in obstetrics and gynecology, are you considering a career in obstetrics, gynecology or both:

	Obstetrics	Gynecology	Both
First choice			
Second choice			
No			

6. Following your rotation in obstetrics and gynecology, do you consider opportunities to train in obstetrics and gynaecology to be:

Good	
Limited	
Do not know	

7. To what extent do you consider the following to be merits of obstetrics as a career (please tick any that apply):

Exciting	
Interesting	

Fulfilling	
Rewarding	
Challenging	
Variety	
Training/career	

8. To what extent do you consider the following to be merits of gynecology as a career (please tick any that apply):

Exciting	
Interesting	
Fulfilling	
Rewarding	
Challenging	
Variety	
Training/career	

9. To what extent do you consider the following to be demerits of obstetrics as a career (please tick any that apply):

Demanding	
Boring	
Stressful	
Incompatible with family life	
High risk	
Fear of litigation	
Long hours/incompatible with family	
Narrow	
Broad	

10. To what extent do you consider the following to be demerits of gynecology as a career (please tick any that apply):

Demanding	
Boring	
Stressful	
Incompatible with family life	
High risk	
Fear of litigation	
Long hours/incompatible with family	
Narrow	
Broad	

11. Would any of the following impact on your decision to pursue a career in obstetrics:

	To a great extent	Somewhat	Very Little	Not at all
Desire to avoid high-risk procedures				
Desire to avoid high-risk patients				
Fear of being sued				
Fear of being reported to/sanction by the medical council				
Fear of criminal prosecution				
Intensity of workload				
Having to do both obstetrics and gynecology				

12. Would any of the following impact on your decision to pursue a career in gynecology:

	To a great extent	Somewhat	Very Little	Not at all
Desire to avoid high-risk procedures				
Desire to avoid high-risk patients				
Fear of being sued				
Fear of being reported to/sanction by the medical council				
Fear of criminal prosecution				
Intensity of workload				
Having to do both obstetrics and gynecology				

13. Would you fear being sued more in obstetrics than any of the following specialties (please tick any that apply):

Anaesthesia	
Cardiology	
Cardiothoracic Surgery	
Chemical Pathology	
Clinical Microbiology	
Clinical Pharmacology & Therapeutics	
Dermatology	
Diagnostic Radiology	
Endocrinology	
Emergency Medicine	
Gastroenterology	
Genito-Urinary Medicine	
Geriatrics	
General Internal Medicine	
General Surgery	
General Practice	
Haematology	
Histopathology	
Immunology	
Infectious Diseases	
Medical Oncology	
Nephrology	
Neurology	
Neurosurgery	
Occupational Medicine	
Medical Ophthalmology	
Otolaryngology	
Paediatrics	
Paediatric Surgery	
Palliative Medicine	
Plastic Surgery	
Psychiatry	
Public Health Medicine	
Radiation Oncology	
Rehabilitation Medicine	
Respiratory Medicine	
Rheumatology	
Surgical Ophthalmology	
Trauma & Orthopaedic Surgery	
Urology	

14. Would you fear being sued more in gynecology more than any of the following specialties (please tick any that apply):

Anaesthesia	
Cardiology	
Cardiothoracic Surgery	
Chemical Pathology	
Clinical Microbiology	
Clinical Pharmacology & Therapeutics	
Dermatology	
Diagnostic Radiology	
Endocrinology	
Emergency Medicine	
Gastroenterology	
Genito-Urinary Medicine	
Geriatrics	
General Internal Medicine	
General Surgery	
General Practice	
Haematology	
Histopathology	
Immunology	
Infectious Diseases	
Medical Oncology	
Nephrology	
Neurology	
Neurosurgery	
Occupational Medicine	
Medical Ophthalmology	
Otolaryngology	
Paediatrics	
Paediatric Surgery	
Palliative Medicine	
Plastic Surgery	
Psychiatry	
Public Health Medicine	
Radiation Oncology	
Rehabilitation Medicine	
Respiratory Medicine	
Rheumatology	
Surgical Ophthalmology	
Trauma & Orthopaedic Surgery	
Urology	

15. Do you think that the staff you have worked in obstetrics with during your rotation fear being sued?

Always	
Very often	
Sometimes	
Rarely	
Never	

16. Do you think that the staff you have worked with in gynecology during your rotation fear being sued?

Always	
Very often	
Sometimes	
Rarely	
Never	

17. The current medico-legal and regulatory culture can have a significant impact on physicians, both personally and professionally. Defensive medicine occurs when medics order unnecessary 'tests, procedures, or visits, or avoid certain high-risk patients or procedures'. Defensive medical practices are employed in an attempt to avoid medical negligence litigation, and/or complaints. Did you see evidence of defensive medicine during your rotation in obstetrics and gynecology?

Never	
Seldom	
Occasionally	
Frequently	

18. Did you see evidence of defensive medicine during any other rotation?

	Never	Seldom	Occasionally	Frequently	Not yet completed
General Practice/Primary Care					
Medicine					
Obstetrics & Gynaecology					
Paediatrics					
Psychiatry					
Surgery					

19. Has you experience in obstetrics and gynecology made it more or less likely that you will pursue a career in:

	More likely	Less likely	No change
Obstetrics			
Gynecology			
Obstetrics and Gynecology			
